# 

**Published:** 1884-02

**Authors:** 


					﻿Vol IV.
FEBRUARY, 1884.
NO. 2.
THE
HOMEOPATHIC PHYSICIAN*
A MONTHLY JOURNAL OF MEDICAL SCIENCE.
“ Seek the Truth: come whence it may, cost tvhat it will."
IE. X LEM JVL. D.,
EDITOR.
CONTENTS :
(See inside of Cover.)
PHILADELPHIA:
No. 2109 CHESTNUT STREET.
NEW YOUK:
A. T CHATTERTON, PUBLISHING COMPANY, Publisher’s Agents.
luOisr J3O its
ALFRED HEATH & CO., Sole Agents foe Great Britain,
114 Ebury Street, Eaton Square.
Yearly Subscription, $2.50, invariably in advance.
Entered at the Philadelphia Post-Office as Second-Class Mail Matter.
				

